# Designing for Motivation, Engagement and Wellbeing in Digital Experience

**DOI:** 10.3389/fpsyg.2018.00797

**Published:** 2018-05-28

**Authors:** Dorian Peters, Rafael A. Calvo, Richard M. Ryan

**Affiliations:** ^1^School of Electrical and Information Engineering, University of Sydney, Sydney, NSW, Australia; ^2^Institute for Positive Psychology and Education, Australian Catholic University, Sydney, NSW, Australia

**Keywords:** HCI, user experience, wellbeing, self-determination theory, design, motivation, engagement

## Abstract

Research in psychology has shown that both motivation and wellbeing are contingent on the satisfaction of certain psychological needs. Yet, despite a long-standing pursuit in human-computer interaction (HCI) for design strategies that foster sustained engagement, behavior change and wellbeing, the basic psychological needs shown to mediate these outcomes are rarely taken into account. This is possibly due to the lack of a clear model to explain these needs in the context of HCI. Herein we introduce such a model: Motivation, Engagement and Thriving in User Experience (METUX). The model provides a framework grounded in psychological research that can allow HCI researchers and practitioners to form actionable insights with respect to how technology designs support or undermine basic psychological needs, thereby increasing motivation and engagement, and ultimately, improving user wellbeing. We propose that in order to address wellbeing, psychological needs must be considered within five different spheres of analysis including: at the point of technology *adoption*, during interaction with the *interface*, as a result of engagement with technology-specific *tasks*, as part of the technology-supported *behavior*, and as part of an individual's *life* overall. These five spheres of experience sit within a sixth, *society*, which encompasses both direct and collateral effects of technology use as well as *non*-user experiences. We build this model based on existing evidence for basic psychological need satisfaction, including evidence within the context of the workplace, computer games, and health. We extend and hone these ideas to provide practical advice for designers along with real world examples of how to apply the model to design practice.

## Introduction

### The impact of technologies on psychological wellbeing

Every technology can deliberately or inadvertently impact psychological wellbeing. As a simple example, consider the nuanced impacts emerging from the instant connectivity made possible by smartphones. Kushlev and Dunn (2015) demonstrated that the number of times a day people could check email increased stress levels, while other studies show that the mere presence of a mobile phone diminishes the quality of face-to-face interaction (Przybylski and Weinstein, 2013; Misra et al., 2016).

Beyond these unintended effects, technologies can also be consciously designed to enhance or regulate people's emotions (Norman, 2005) and over the last 15 years interaction designers have shifted their focus from mere usability to also making products enjoyable and engaging, generally with the goal of increasing usage. However, factors such as engagement and enjoyment do not necessarily contribute to sustainable wellbeing. Indeed, as studies in video games (Rigby and Ryan, 2011) and media consumption (Hefner and Vorderer, 2017) confirm, too much engagement can crowd-out healthy activities to the detriment of overall wellbeing. Thus a larger question remains: How can technology be designed to support wellbeing that encompasses more than just immediate hedonic experience, but also its longer-term *eudaimonia*, or true flourishing? (Ryan and Deci, 2001, 2017; Sirgy, 2012).

### Design for wellbeing in HCI

A desire to design for deeper meaning, happiness, and human flourishing has gained momentum in HCI over the past 5 years, and both researchers and practitioners have struggled to bridge this new impetus to clear actionable practice.

Among the contributions to this area is work on Positive Technologies (Riva et al., 2012), Experience Design (Hassenzahl, 2010), Positive Design (Desmet and Pohlmeyer, 2013), and Positive Computing (Calvo and Peters, 2014). At the broadest level, Positive Technologies takes from positive psychology and argues for the benefits of using technology to influence the (1) affective quality, (2) engagement/actualization, and (3) connectedness of experience. Examples of positive technologies have generally been virtual reality environments and other forms of software design as interventions for mental health and wellbeing. Positive Design on the other hand has focused on how the design of any artifact, built environment or service might foster flourishing. Desmet and Pohlmeyer's framework for wellbeing requires that a product be designed for virtue, pleasure and/or meaning, where none of these components interferes with the others (Desmet and Pohlmeyer, 2013). Hassenzhal has proposed an experience-focused approach centered on “fulfilling psychological needs” (including autonomy, popularity, stimulation and others) as a method for inscribing meaning and happiness into products (Hassenzahl, 2010). He proposes doing so by uncovering “experience patterns” in human activities that distil the essence of certain need-fulfilling practices. Finally, as part of Positive Computing, Calvo and Peters (2014) have focused on wellbeing-supportive design for all technology by targeting wellbeing determinants (i.e., self-awareness, compassion, gratitude, motivation, etc.) and by leveraging the research and measures for these constructs for design and evaluation.

The four approaches described above (Riva et al., Desmet and Pohlmeyer, Hassenzhal, and Calvo and Peters) provide pathways to inspiring design based on psychological factors shown to contribute to wellbeing. In addition, other combined editorial works such as (Calvo et al., 2016; Villani et al., 2016) help bring together frameworks and empirical evidence helpful to advancing the field.

However, there remains a substantial gap between existing frameworks and immediately actionable design practices. For example, a library of validated “experience patterns” as Hassenzhal's work points to, has yet to be developed. Clear design features relating to wellbeing determinants, pleasures, virtues or meaning (as Positive Computing and Positive Design recommend) have yet to be identified. Most importantly, perhaps, in light of urgent concerns with technology addiction, none of the frameworks provides help or guidance on how design can disentangle engaging experiences that are *healthy* from engaging experiences that are *addictive*. In other words, the design community has made important headway in shaping what we believe to be the next era in human-centered technologies, but more bridge-building is necessary before the practice of wellbeing-supportive design can be robustly deployed across the industry.

The field requires a model based on methodologically sound approaches that can support hypotheses which can be tested experimentally. This model, and the studies it would support, would allow for experience patterns to be developed, design strategies to be identified and unhealthy positive experiences to be differentiated from healthy ones. In this paper we propose a candidate for such a model of wellbeing-supportive design along with practical methods for working with that model.

### The three keys to engagement, motivation and wellbeing

The core elements in our solution to designing for wellbeing leverage *Self-Determination Theory* (SDT; Ryan and Deci, 2000b, 2017) which provides a mature and empirically-validated approach to examining factors that promote sustained motivation and wellbeing. Although a nuanced theory, in its broadest strokes, SDT identifies a small set of basic psychological needs deemed *essential* to people's self-motivation and psychological wellbeing (Ryan et al., 2013), and whose neglect or frustration is associated with ill-being and distress. These basic needs are:

- **Autonomy** (feeling agency, acting in accordance with one's goals and values),
- **Competence** (feeling able and effective),
- **Relatedness** (feeling connected to others, a sense of belonging).

These three factors are a sort of minimum common denominator, which come with the widest research evidence available (see Ryan and Deci, 2017 for a review) to explain causal relationships between independent variables (design features) and dependent variables (wellbeing, motivation and engagement measures).

This differs from the approach taken by other authors. For example, Hassenzhal and colleagues incorporate “popularity” as a psychological need (Hassenzahl et al., 2010) however, we argue that popularity is sometimes a desired *outcome* mediated by the basic psychological needs for relatedness and competence and not a universal core need in and of itself. Likewise, the wellbeing determinant, “compassion” which we've elaborated on in previous work (Peters and Calvo, 2014), is also a wellbeing-supportive *outcome* which is itself mediated by the three basic needs (largely relatedness, but also autonomy and competence which differentiate compassion from empathic distress (Peters and Calvo, 2014).

We are certainly not suggesting there is no value in using constructs such as popularity or compassion to inform design. Nor are we attempting to reduce the totality of human psychological experience to three constructs. We are simply highlighting that these three are the most rigorously shown to be essential and predictive of wellbeing and other desired HCI outcomes, and therefore most critically important to assess within HCI contexts. Specifically, SDT defines the term “basic psychological need” very strictly as those satisfactions that:

- are inherently rewarding/motivational.
- when satisfied lead to flourishing.
- when frustrated lead to negative experience.
- function across diverse cultures and developmental stages.

At first blush this may seem like a lot to attribute to three constructs, but a more thorough exploration of them reveals a depth and clear link to more commonly articulated concepts like meaning or happiness. More importantly, this claim is based, not on opinion, but on four decades of empirical research systematically demonstrating these specific three factors to be the most predictive and reliable mediators of motivation, engagement and wellbeing. A survey of the literature is out of scope for this paper, but Ryan and Deci (2017) and Vansteenkiste and Ryan (2013) provide comprehensive reviews.

In addition, several meta-analyses aggregate the results of multiple studies to provide robust evidence for these needs within various domains. For example, Ng et al. (2012) aggregated data from 184 studies exploring SDT constructs for behavior change in health. A meta-analysis by Hagger and Chatzisarantis (2009) combined the results of 34 studies of the Theory of Planned Behavior (TPB) and SDT and provide cumulative empirical evidence of how SDT predicts intentions and behavior in the TPB. A meta-analysis by Chatzisarantis et al. (2003) used 21 studies to explain motivation and SDT constructs in the context of exercise, sport, and physical education. Gagné and Deci (2005) analyzed the literature on how SDT explains the interaction of intrinsic and extrinsic motivation in the workplace.

It is also important that the basic needs defined by SDT are: measurable, intrinsically rewarding, and always safe targets for design because there is no point at which you “overfill” on them (as opposed to, for example, stimulation as posited by Hassenzahl et al., 2010). For example, with regard to autonomy, people cannot have too much volition—feel “too willing” to act (they want to feel as autonomous as possible). People cannot feel *too* competent (yes, one can be bored, but not too effective as in “I wish I were less competent at this”). Finally, one cannot feel too much relatedness—even if one can get too much meaningless or unwanted social stimulation. Understanding these basic needs is important for design because it represents a path in which experiences of inherent import to users can be addressed and without great risk of overdoing it.

### Basic psychological needs as effective proxies for wellbeing-supportive design

There are many constructs that describe the positive elements of human psychological experience (serendipity, fun, praise, gratitude, etc.) and any of these can be very useful to design for ideation and insight. However, by distilling our focus to just the three basic psychological needs that have been consistently and cross-culturally shown to mediate wellbeing, we are handed the controllers, so to speak, of wellbeing-supportive experience.

While the secrets to engagement, motivation and wellbeing have often appeared to reside inside a black box, what research shows is that it is the basic needs that are in that box. In other words, if you increase autonomy then engagement will improve, if you increase competence then motivation will increase, and if you increase relatedness then wellbeing will be enhanced–these needs become the controllers we tweak and adjust to iterate on and improve experience. In other words, basic needs are the mediating variables between product and well-being, and thus can be used as proximal criteria for adjusting design (making possible the “usable evidence” called for by Klasnja et al., 2017).

For example, SDT has been used to develop “personas” of digital coaches (Jansen et al., 2017). It can also be used during testing, for example, to evaluate feedback from a wearable device in order to optimize product satisfaction. Does the device provide feedback that increases feelings of mastery (enhancing competence) or does the feedback provided feel like empty praise or meaningless numbers? Does the device offer meaningful choices (enhancing autonomy)? Do features that connect users actually increase relatedness? In this way, the specific features of an interface can be measured against psychological need satisfaction and adjusted accordingly with resulting improvements to user experience, engagement and wellbeing.

### Links to behavior change

Basic psychological needs are not new to HCI. They have already been applied, but almost exclusively as a model of motivation to enable behavior change (i.e., Hekler et al., 2013). In contrast, the literature linking psychological needs to *wellbeing* or sustained *engagement* is less well-known within the HCI community and therefore fewer links have been made.

This paper answers a call extended by Hekler et al. (2013) for “behavioral scientists and HCI researchers to work together on the design of behavior change technologies.” Specifically they advocate drawing on theory to “make decisions about *which* functionality to support and *how* to implement such functionality.” In their paper, which provides guidance to HCI researchers on the use of behavioral theories, they discuss behavior change models such as TPB (Ajzen, 1991), Self-Efficacy Theory (Bandura, 1996; Schunk and Usher, 2012), and SDT (Ryan and Deci, 2000b, 2017). These are all large-scale theories that generalize to multiple contexts (i.e., meta-models), but which can often be hard to apply in HCI with much resolution. Herein, we provide tools to make this application, regarding self-determination theory (SDT), far more straightforward. Our SDT-based model *Motivation, Engagement, and Thriving in User Experience (METUX)* is described below.

### Background summary and walkthrough

In summary, SDT identifies three basic needs, the satisfaction of which are known to increase three primary desired outcomes of user experience: motivation, engagement and wellbeing. Therefore, through conscious design and testing, designers can focus on supporting these basic needs through the functions, features and contents of their technologies in order to improve user experience and wellbeing. Evidence for this impact and the practical links to design decisions are included in this paper.

We first introduce relevant SDT constructs and how they can be adapted holistically to the technology design context. Then we present METUX, a model that can be used for the evaluation and iterative design of technologies in order to optimize engagement, motivation and wellbeing. We elaborate on motivational design in a technology context and provide measures that can be used to evaluate designs for psychological needs in practice.

## Critical concepts for a model of wellbeing-supportive design

### The importance of differentiating spheres of impact

**Figure 1:**
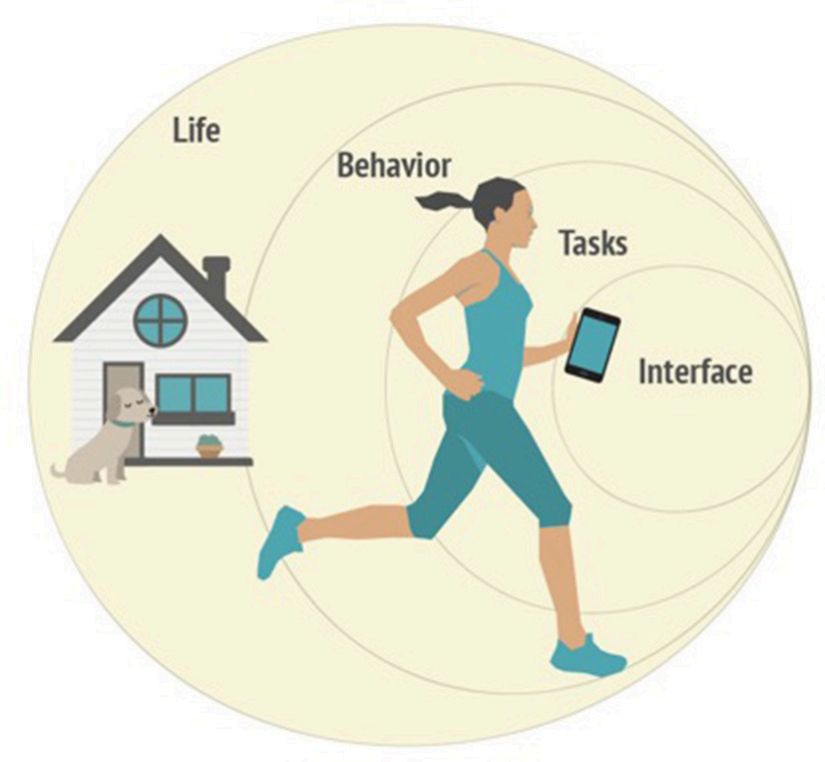
User Experience of wellbeing – Spheres of Experience within which technology can influence wellbeing.

Calvo et al. (2014) explored ways in which autonomy can be influenced by technology within various spheres of experience. This work highlighted the necessity for specificity about the levels at which need satisfaction can take place. For example, “autonomy-support” could equally refer to the addition of customization to software, or to the extent a self-driving car increases autonomy in the daily life of someone who is vision-impaired. We posit that it is helpful to think about how a technology influences wellbeing within at least four different spheres of experience: (1) As part of interacting with the technology via its *interface*, (2) As part of engaging with technology-enabled *tasks* (e.g., self-tracking) (3) In relation to the overarching technology-supported *behavior* (e.g., exercise) 4. As part of a user's overall *life* (See Figure 1).

Acknowledgment of these differing spheres of experience is essential if we are to avoid creating technologies that are need-satisfying at one level but need-frustrating at another (i.e., addictive). Nevertheless, this acknowledgment has been largely missing from the literature on design for human factors. The conceptions about HCI as a discipline (Long and Dowell, 1989) have often limited research to what goes on at the interface level, even arguing that some spheres are beyond the bounds of HCI (Siek et al., 2014). Others like Smith et al. (2014) have instead valued the importance of considering impact on a “value-chain” of proximal intermediate and distal effects. No matter how wide the purview of HCI *per se*, design for wellbeing is an interdisciplinary endeavor and can therefore not be bound by disciplinary limits if we are to design holistically for thriving. Our model, therefore includes consideration of the different spheres of experience within which psychological needs can be influenced and we describe these in greater detail in relation to the model in section A model for Motivation, Engagement, and Thriving in the User Experience (METUX).

### Basic psychological needs in context

Before discussing the METUX model it is worth taking a brief look at more complete definitions of the three basic psychological needs and how they have already been used in service of HCI research. The first and most widely studied within technology domains is autonomy.

#### Autonomy

The term autonomy literally means to be governed by the self and refers to a sense of willingness, endorsement or volition in acting (Ryan and Deci, 2017). Autonomy should not be confused with merely doing things independently or being in control; rather when people act with autonomy, they act with high willingness and in accordance with their personal goals and values, connecting autonomy to meaning & purpose. An individual can very willingly relinquish control or embrace interdependence. As a result of autonomous experience, an individual's quality of behavior and performance is higher and they experience greater wellness. A growing understanding of the importance of autonomy has lead to a radical shift within healthcare and a parallel change on the horizon in engineering. Where in the past, doctor-patient relationships left little room for patient agency, biomedical ethicists (Beauchamp and Childress, 2001) now consider deference to patient autonomy as a guiding principle.

Within engineering, the vast majority of research has focused on the design of autonomous *systems*, particularly robots and vehicles, rather than on supporting autonomous *humans* (Baldassarre et al., 2014). More recently however, the Institute of Electrical and Electronics Engineers (IEEE) has developed a charter of ethical guidelines for the design of autonomous systems that places *human* autonomy and wellbeing center-stage (Chatila et al., 2017). One of our aims within this paper is to assist technology creators in this quest to respect and support human autonomy as part of overall psychological need satisfaction in future technology design.

Friedman (1996) identified four aspects of software systems than can support or hinder user autonomy (i.e., system capability and complexity, misrepresentation, and fluidity) but focused on the direct impact of the system's use (what we would refer to as the interface sphere) and not the broader impact on other aspects of a person's life.

Design for autonomy is very familiar to game designers (Ford et al., 2012) and has been shown to predict measures of presence and intuitive controls (Ryan et al., 2006). Devices that offer options and choices over use, and do not in turn demand actions from users without their assent, enhance feelings of autonomy. Personalization also creates a sense of ownership and choice beneficial to autonomy (Ryan and Rigby, 2018). Ryan et al. (2006) showed how perceived autonomy in video games can lead to game enjoyment, preferences, and short-term wellbeing. Furthermore, Peng et al. (2012) tested an interactive exercise game in which an autonomy-enhancement feature was turned “on” and compared to an “off” condition. The feature inclusion significantly affected game enjoyment, motivation for future play and overall game ratings. Most relevant is that the relationship between the design feature and engagement was mediated specifically by autonomy in expected ways, consistent with SDT.

Beyond the sphere of the user interface, technologies can also facilitate greater autonomy within daily life by removing obstacles or augmenting capabilities, allowing people to pursue self-determined goals more fluently. For example, assistive technologies, productivity tools or health management apps, can all increase autonomy in relation to daily behaviors.

Finally, there is the potential for technologies to foster autonomy as an overarching characteristic of psychological development and flourishing. For examples, some technologies such as educational, health or behavior change tools, might help users develop a greater sense of autonomy in their lives generally and to more effectively realize their personally held values. In sum, there are many opportunities within various spheres for technologies to be autonomy-supportive and research shows that making them so, will foster engagement, motivation and wellbeing.

#### Competence

Competence, or feeling capable and effective, is the second psychological need identified by SDT. There are certain factors that have been shown to enhance a sense of competence including optimal challenge, positive feedback and opportunities for learning. These will be familiar to usability engineers as all usability heuristics can be explained by the needs for competence and autonomy. In the sphere of video games, for example, Rigby and Ryan (2011) detail how the intuitive design of controls, and the density and clarity of feedback all impact engagement via increased competence. In fact, controversies over the importance of difficulty and novelty in games (Lomas et al., 2017) can be better understood as competence issues. Both “difficulty” and “novelty” are only important to engagement to the extent to which they provide competence satisfactions. A game that is too easy stops providing them, as does one that is too hard. Novelty (such as new level designs or new rewards) is also engaging to the extent that it provides new opportunities for competence satisfactions (new designs and features promise new opportunities for learning and mastery).

Illustratively, Peng et al. (2012) in the exergame experiment mentioned above, manipulated a competence-enhancement condition based on dynamic difficulty. Specifically, the program featured an automated system to create optimal challenges based on player performance, whereas in the “off” condition challenge levels remained relatively constant. Decreased game enjoyment was mediated by a shift in competence satisfactions. This work demonstrates how design features might be iterated with respect to their impacts on need satisfactions toward improving the user experience.

#### Relatedness

Relatedness is described as a sense of belonging and connectedness to others and it is core to most, if not all, theories of wellbeing (Baumeister and Leary, 1995). Research has even linked positive relationships to greater longevity more powerfully than diet and exercise (Kasser and Ryan, 1996). Yet not all social interactions help people feel a greater sense of belonging or connectedness. Many app features and communication devices can even frustrate relatedness with subsequent impacts on engagement and wellbeing. Moreover, such affects can occur as a result of apparently small details and in ways that are surprising (e.g., Hudson et al., 2015 found that emoticons influence Facebook jealousy).

Considering the explosion of new social media technologies, support for relatedness arguably defines a category of digital experience that shapes our generation. What SDT provides us with are assessments of relatedness against which specific features of devices (e.g., video chats, cooperative features, emoticons, nudges, etc.) can be tested to ensure that they are meaningful, satisfying, and lead to genuine relatedness, rather than the mere semblance of connection, hurtful interactions or social isolation (e.g., Sheldon et al., 2011).

Significant qualitative differences between different types of technology-enabled social connection have already been suggested by a number of studies, most notably, observational studies on Facebook use. For example, (Burke et al., 2010) found that directed communication between pairs (i.e., wall posts, comments, and “likes”) is associated with greater feelings of bonding social capital and lower loneliness, whereas, users who engage in mere browsing of friends' content (i.e., status updates, photos, and friends' conversations with other friends) report reduced social capital and increased loneliness. Furthermore, they point to how these findings could inform design decisions, specifically “enhancements for fostering communication over passive engagement.” Furthermore Grieve and Watkinson (2016) showed that in Facebook, only authentic self-representation was associated to wellbeing, while lack of authenticity was related to stress and lower wellbeing which suggests that a design promoting authentic self-representation may have better wellbeing outcomes within these technologies.

There has also been suggestion as to how design might be directed to support constructs such as empathy and compassion (Belman and Flanagan, 2009; Peters and Calvo, 2014). Principles posited in both of these works suggest that the satisfaction of psychological needs mediate these constructs as well. In these, as well as in the Facebook experiments, if we were applying the model described herein, established measures of relatedness would be used to determine the impact of various designs and to help pre-empt inadvertent harm.

In short, because our relationships are increasingly mediated by technology, and because technology experience is increasingly social, models and measures of relatedness stand to contribute to both the literature on wellbeing and to the future of technology design.

### The importance of motivation type (autonomous vs. controlled)

An additional contribution of SDT to technology design is the insight that the value of motivation (in terms of its ability to contribute to wellbeing) depends strongly on how *autonomous* (v. extrinsically-controlled) it is. In other words, someone can be highly motivated in ways that are highly controlled and that don't foster wellbeing (e.g., by threat of punishment.) In contrast, extrinsic motivation that is highly *autonomous* is highly effective and does contribute to wellbeing (with outcomes similar to those of intrinsic motivation (Ryan and Deci, 2000a, 2017).

Deci et al. (1999) in a meta-analysis of 128 studies, confirmed that rewards contingent to engagement, completion, and performance undermined intrinsic motivation. Positive feedback instead enhanced free-choice behavior and interest. A meta-analysis by Ng et al. (2012) within the healthcare context, confirmed that autonomous motivation supports more effective and lasting behavior change. Specifically, an autonomy-supportive health care climate positively predicted need satisfaction which, in turn (together with autonomous motivation) predicted better health outcomes.

**Figure 2:**
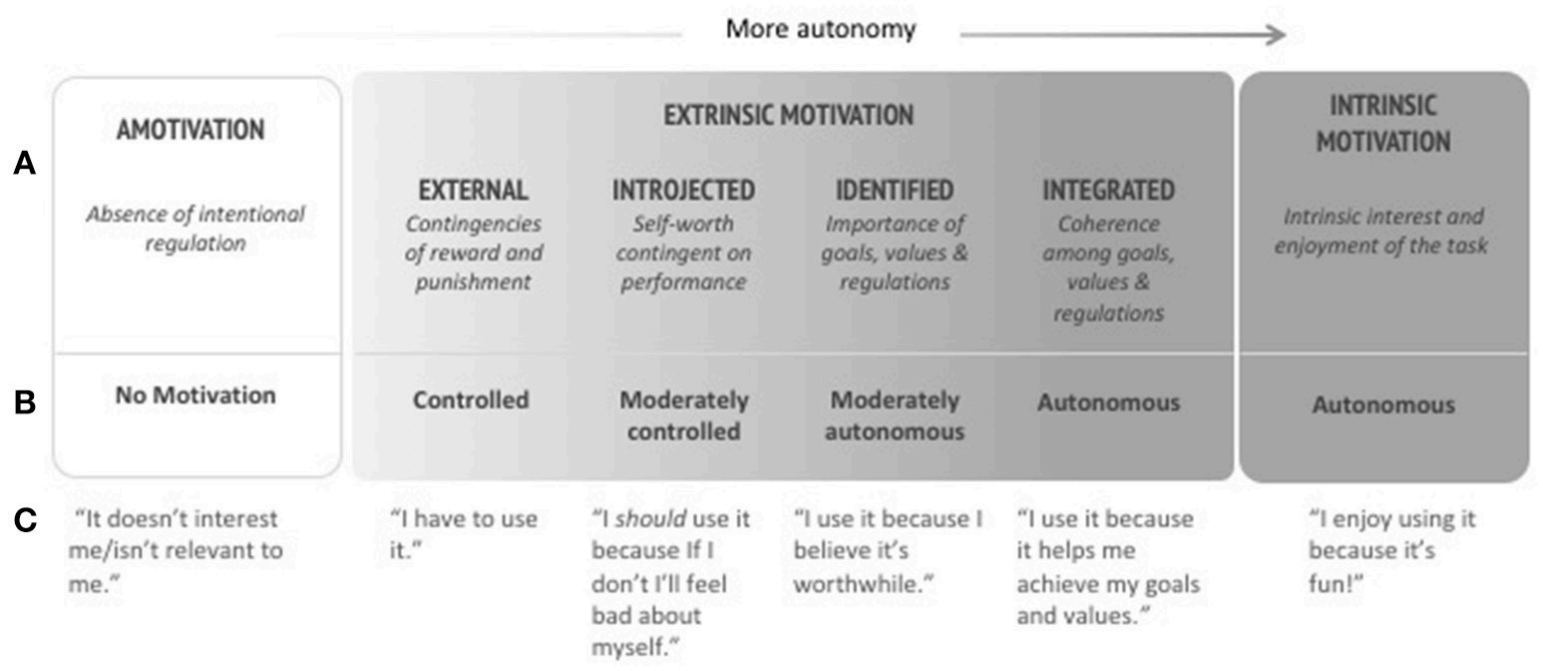
Taxonomy of Human Motivation; **(A)** Type of regulation, **(B)** Type of motivation, and **(C)** Examples translated to the user experience context (Adapted from Ryan and Deci, 2000a).

Figure 2 shows that intrinsic and extrinsic forms of motivation can be placed on a continuum from controlled to autonomous. Here we have redrawn Ryan and Deci's original model (Ryan and Deci, 2000a) adding a “User experience” row in order to show how the model applies within the technology context (see Figure 2). Controlled extrinsic motivation involves a sense of pressure or obligation and often includes extrinsic rewards or penalties (Ryan and Deci, 2000a), while highly autonomous extrinsic motivation is close in quality to intrinsic motivation with regard to its ability to foster wellbeing and positive outcomes. In other words, even when something isn't fun (intrinsically motivating), we can be very meaningfully motivated to engage with it when our motivation is highly autonomous.

A plethora of new technologies promise to motivate people to engage in healthy behaviors, but as SDT has shown, the way behaviors are initiated and maintained (autonomously or via controlling methods) will have a significant impact. More recently, researchers have begun exploring how *physical* health apps can also support *psychological* wellbeing. For example, Karapanos et al. (2016) explored how commercial wearable activity trackers mediate meaningful experiences in everyday life. While most commercially available trackers employ competition as the primary mode of social exchange and motivation, their study showed that tracking involves much more nuanced socially motivated experiences, including a sense of belonging, social support, and bonding.

Having specified the various categories of motivations upon which our discussion draws, we can now describe our model for wellbeing-supportive design.

## A model for motivation, engagement, and thriving in the user experience (METUX)

A number of existing evidence-based models inform and set a precedent for the need-satisfaction-based model we propose. For example, the SDT model of health behavior change (Ryan et al., 2008) shows how a combination of environmental and individual determinants can support or hinder need satisfaction within the health context. Furthermore, the model predicts how need satisfaction will also have a positive impact on mental and physical health outcomes.

Similarly, the SDT model of video game engagement is focused on what has been called the PENS (*Player Experience of Need Satisfaction*; Ryan et al., 2006). In this model both game contents (e.g., narrative and story) and features (e.g., open world, goal choices, dialogue boxes, etc.) all affect satisfactions of autonomy, competence and relatedness during play, in turn predicting enjoyment and sustained engagement. The PENS framework is readily tested using assessments of need satisfaction such as those employed by Ryan et al. (2006) which can be broadly applied (Przybylski et al., 2010).

**Figure 3:**
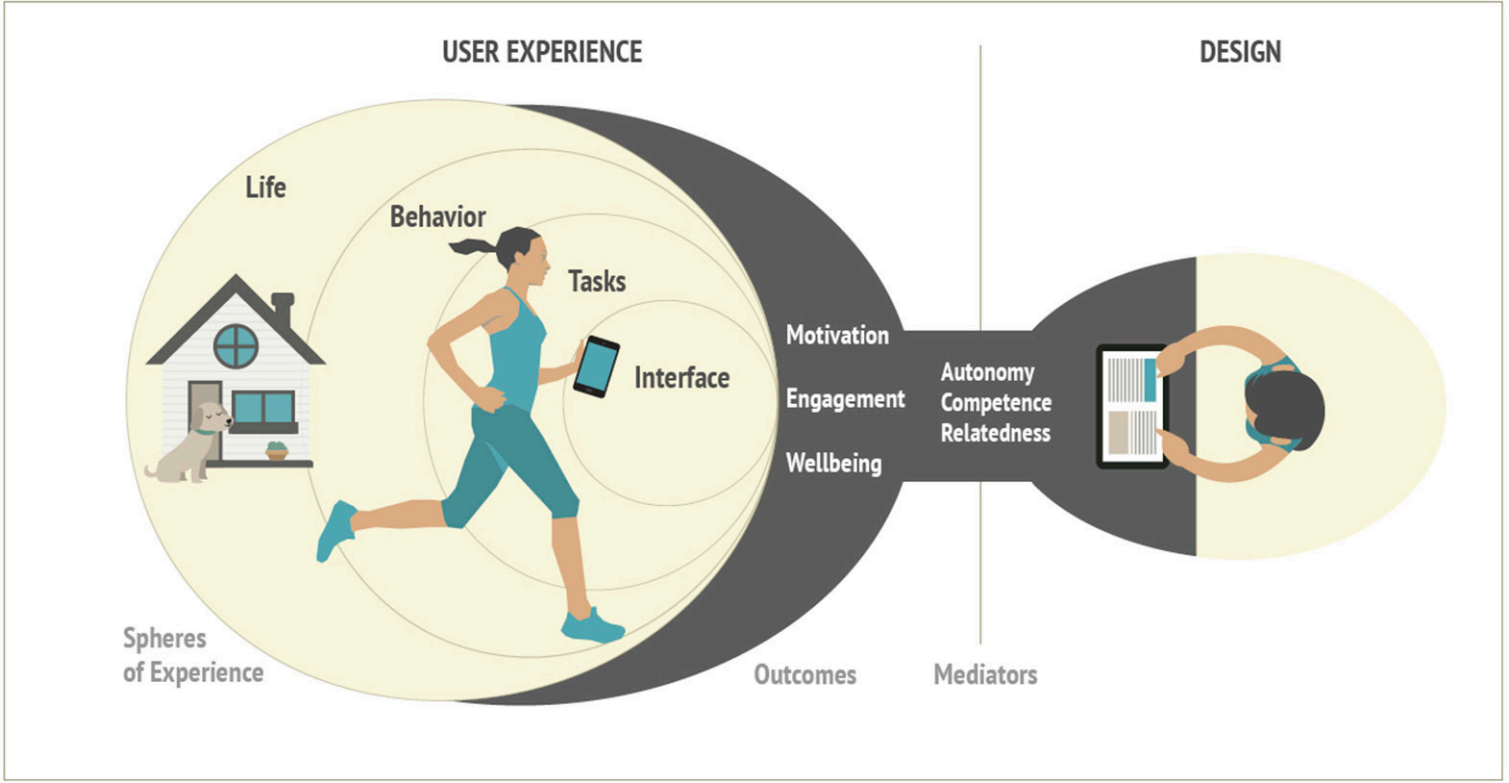
METUX model diagram - The basic psychological needs of autonomy, competence and relatedness mediate positive user experience outcomes such as engagement, motivation and thriving. As such, they constitute specific measurable parameters for which designers can design in order to foster these outcomes within different spheres of experience.

Drawing on the evidence and previous work in health, video games and other domains including workplace, education, and sport (see Ryan and Deci, 2017 for a review), our model (Figure 3 and Table 1) applies existing evidence to describe and predict the impact of technologies on motivation, engagement and wellbeing based on psychological needs satisfactions.

**Table 1:** METUX model in detail, including measures.

**Sphere of experience**	**Psychological needs (mediators) in context**	**Evaluation measures**	**Desirable outcomes**
**Adoption**	To what extent is technology adoption autonomously motivated? To what extent does a potential user anticipate they will be competent at using it?	ACTA^i^	Adoption (i.e., purchase, download)
The decision-making experience between becoming aware of a new technology and acquiring it.			
**Interface**	To what extent does direct interaction with the technology (i.e., via the user interface) support psychological need satisfaction?	TENS-Interface^i^	Engagement (with technology) Usability User satisfaction
The experience of interacting with a technology via its interface during use.			
**Task**	To what extent does engagement in technology-specific tasks support psychological need satisfaction? (e.g., step tracking, text chatting)	TENS-Task^i^	Engagement (with task) User satisfaction
The experience of engaging in a technology-specific task.			
**Behavior**	To what extent does the technology improve psychological need satisfaction with respect to the behavior that the technology is intended to support? (e.g., exercise, managing a chronic disease, communicating with friends, speaking a second language.)	Assessments of psychological need satisfaction in relation to behavior (e.g., PNSES^ii^ for exercise) Assessments of behavior-specific outcomes (e.g., BMI measure for exercise)	Engagement (with behavior) Satisfaction (with behavior) Behavior-specific outcomes (e.g., weight-loss, symptom control) Experience of wellbeing during behavior.
The experience of engaging in a behavior (that a technology is intended to support).			
**Life**	To what extent does the technology influence the user's experience of psychological need satisfaction in their life overall?	TENS-Life^i^ BPNS^iii^ Other validated measures of flourishing	Increased life satisfaction, wellbeing, thriving/flourishing.
An individual's overall experience of life including all that is outside or beyond the technology.			
**Society**	To what extent does the introduction of the technology impact on societal wellbeing?	Population measures such as the FS^iv^	Increased measures of societal wellbeing.
The experiences of all members of a society beyond the users of a technology.			

i *ACTA, TENS-Interface, TENS-Task and TENS-Life are introduced in section 4*.

ii *PNSES, Psychological Need Satisfaction in Exercise Scales (Wilson et al., 2006)*.

iii *BPNS, Basic Psychological Need Satisfaction scale (Deci and Ryan, 2000; Gagné, 2003)*.

iv FS, Flourishing Scale (Diener et al., 2009)

The table below lists six separable spheres of experience that can be influenced by technology design. Figure 3 illustrates the relationship between design for psychological need satisfaction and engagement, motivation and wellbeing within each sphere of experience. The first five spheres of experience identified by the model refer to elements of the *individual* user experience upon which a technology can have an impact.

*Adoption* is not pictured in the diagram because of its peripheral role preceding actual use, and neither is the broadest sphere, *society* owing to its position beyond the individual user experience. Society is the one sphere that goes beyond the user to encompass *non*-user experiences, and collective and collateral effects.

It is worth noting that the boundaries between these spheres is merely conceptual and examples of overlap and interrelation exist. The point is not to overemphasize boundaries but to provide a way of organizing thinking and evaluation in a way that can address contradictory parallel effects (i.e., a technology can support psychological needs at one level while undermining them at another). Each of these spheres is described in greater detail below, while descriptions of evaluation measures follow in the next section. We also provide three examples of how the spheres can provide a valuable framework for the analysis of diverse technologies (Table 2).

**Table 2:** Examples of three diverse technologies through the lens of the METUX model.

**Sphere of experience**	**Wearable fitness device**	**Chronic illness support App**	**Language learning online course**
Adoption	Purchasing the device. *[ACTA]*	Downloading the app *[ACTA]*	Enrolling in the course *[ACTA]*
Interface	Controls, navigation, information display and aesthetics of the device. *[TENS-Interface]*	Controls, navigation, information display and aesthetics within the app *[TENS-Interface]*	Controls, navigation, information display and aesthetics on the site *[TENS-Interface]*
Tasks	Step counting, heart-rate monitoring & session timing *[TENS-Task]*	Symptom tracking, mood tracking & goal-setting *[TENS-Task]*	Vocabulary repetition, text translation, sentence generation *[TENS-Task; SRQ-Learning]*
Behavior	Exercising *[TSRQ-Exercise]*	Managing asthma *[TSRQ* (adaptation for asthma management)*]*	Learning Spanish *[TSRQ* (adaptation for language learning)*]*
Life	Overall wellbeing (influenced by Increased engagement in regular exercise) *[TENS-Life]*	overall wellbeing (influenced by improved asthma control) *[TENS-Life, BPNS]*	overall wellbeing (influenced by ability to communicate in a new language) *[TENS-Life]*
Society	Societal wellbeing (Increase in regular exercise across a population could improve overall societal wellbeing via increased levels of physical and mental health.) *[FS Scale]*	Societal wellbeing (Improved management of asthma could improve overall societal wellbeing via decreased fatalities and increased population health.) *[FS Scale]*	Societal wellbeing (Fluency in an additional language across a population could improve overall societal wellbeing via increased cross-cultural relatedness.) *[FS Scale]*

*Possible measures for evaluation are listed in brackets*.

### ADOPTION—anticipated need satisfaction at the point of adoption

The first level, adoption, begins when a person first becomes aware of a new digital product and ends when he or she acquires and uses it for the first time. The primary outcome of this phase is uptake of the technology. SDT predicts that users will be likely to adopt a new technology to the extent that they are autonomously motivated to do so. Therefore, the primary question is: to what extent is a user's motivation to adopt a technology *autonomous*, that is, willing and aligned with their values and goals (e.g., “I really want to try that app because I think it will help me engage with exercise more”), versus perceived as externally controlled (“my boss is forcing me to download this app”)?

Drawing on the Intrinsic Motivation Inventory (Ryan, 1982), we devised a measure of intrinsic motivation specific to technology adoption (described below). To this measure, we added two perceived competence items, as we hypothesized that a person's willingness to adopt a technology would be influenced by anticipated competence to use it (which can also be framed as perceived ease-of-use). This can be influenced by aesthetics (see “aesthetics-usability effect” Norman, 2004), marketing, a user's prior experience, and their general attitude toward technology adoption.

There seems little scope for any actual increase in relatedness during the adoption phase, therefore, although *anticipated relatedness* can have an influence (e.g., “I will be able to connect with my family better if I use this”) it functions as an autonomous motivator rather than as relatedness itself (anticipated relatedness contributes to autonomous motivation as it aligns with values and goals).

Of course there are a number of existing technology adoption models approaching the problem from various angles, including the Technology Acceptance Model (Davis et al., 1989), based on the Theory of Planned Behavior (Ajzen, 1991), which is used to understand behavior change and persuasion based on “perceived use.” Within the information systems literature “perceived use” has been described as “the degree to which a person believes that using a particular system would enhance his or her job performance” (Davis, 1989). By viewing adoption through the lens of SDT, we can broaden this definition by rephrasing it as “the degree to which a person believes that using a particular system would enhance his or her sense of autonomy, competence or relatedness in any facet of life.”

In our model, we address “perceived use” within the context of motivation which has the added benefit that it allows for compulsory use to be taken into account (people may adopt a technology even if they don't perceive usefulness). For example, someone may autonomously elect to use a video chat app because they anticipate it will increase their productivity (enhance competence) or allow them to connect to their grandchildren (enhance relatedness), both of which are autonomous motivations. On the other hand, someone may be required by their workplace to use it, in which case, they may not perceive any use for it at all but adopt the technology anyway for fear of penalty (externally regulated extrinsic motivation). In other words, an SDT-based approach shifts the focus from the content of perceived use to how autonomously motivated it is.

### INTERFACE—need satisfaction from interaction with the interface

SDT predicts that users will engage with a technology to the extent that interaction with the system satisfies their psychological needs and the primary outcome from need-satisfaction is sustained engagement. One way this manifests is through usability. Poor usability will cause need frustration (to autonomy and competence). Studies by Rigby and Ryan (2011) provide some examples of interface-based need satisfaction that show how variations in video game feature design impact a user's sense of autonomy and competence during play which in turn determine to what extent users engage with and enjoy a technology.

In contrast, relatedness has been less studied with regard to interface interaction, probably because it is not essential to technology engagement (even a digital game of solitaire can be engaging). Relatedness is essential to *wellbeing* but does not have to be served by every technological experience. As such, simply tacking on social features in an attempt to reap the benefits of relatedness is not necessarily advisable, on the one hand because social features don't guarantee relatedness, but also because there are situations in which the quality of the user experience may be diminished if it is shifted from being private to being social at the interface level.

For example, a personal journal or mindfulness app may be far more effective at achieving intended outcomes (honest self-reflection, reduced self-criticism) without the incorporation of social features such as a “share” button. Calvo and Peters (2014) consider how social features applied to mindfulness technologies may make users more likely to compare themselves to others which is antithetical to the goals of mindfulness practice. Interestingly, in this case it could be said that a lack of social features would better contribute to relatedness as it better supports the behavior itself which itself increases relatedness. This increase in relatedness could only be detected *beyond* the level of the interface but is an example of the far-reaching impacts of interface-level choices. These distinctions further demonstrate why unpacking various spheres of experience is helpful for evaluating a design's affect on human psychological needs.

### TASK—need satisfaction from engagement with a technology-enabled task

Every technology has features and functionalities that enable various tasks. For example, a fitness app may allow you to track steps, count calories or read athlete stories. Each of these tasks may be more or less fulfilling of psychological needs; for example, reading athlete stories might make you feel worse or better about yourself depending upon the content of the stories. Likewise, you may find the task of step tracking valuable or frustrating. The step tracking features can be designed in many different ways at the interface level, but the task of step tracking itself is an identifiable activity enabled by the technology.

Finally, a particular task is generally intended to support an overall behavior (e.g., exercise) which brings us to the next sphere.

### BEHAVIOR—need satisfaction related to a technology-supported behavior

With the notable exception of games, most technologies are designed to enable, augment, or enhance some separable overarching behavior. Health apps, for example, may be intended to influence behaviors like exercise, healthy eating, or meditation. Calendaring apps support time-management or event planning while email supports professional or social communication. In other words, you use the technology because it helps you to succeed at something else. You might engage with these behaviors for intrinsic reasons (I exercise because it feels good) or because you're aiming for a separable outcome (I exercise to lose weight). In relation to the tasks sphere described above, the behavior is the overarching activity that a task is intended to support.

The difference between these spheres is important because a technology might support need-satisfying interaction at the interface level (be satisfying to use), and at the task level (completing tasks is satisfying) but may still not necessarily impact need satisfaction in relation to the behavior it's designed to support. For example, a user who adopts a new exercise app may find the app itself engaging but not feel more willingness to exercise as a result of it. Likewise, a user may become very proficient with their calendaring software but it may not make them feel any more autonomous with regard to managing their time. In fact, seeing all events presented in color on one screen may cause them to feel overwhelmed and less in control. Clearly, to truly understand the impact of a technology holistically, measuring need-satisfaction at the interface is not sufficient.

### Life—the link between technology and overall wellbeing

The SDT literature indicates that psychological need satisfaction increases mental and physical health. However, momentary need satisfaction relating to the use of a technology may not be sufficient to affect measurable improvements to individual flourishing. For example, a superbly designed egg timer may improve the cooking experience (allowing the user to feel more autonomous and competent as a cook) and yet this device on its own would not be expected to measurably change a user's overall satisfaction with life. However, one might expect that an effective mindfulness tool would. Therefore, whether a technology goes beyond need satisfaction at the interface, task and behavior spheres, and has a large enough impact to increase overall wellbeing in life, will often depend on what is intended.

The notion of the life sphere is especially useful when assessing technologies that consciously aim to impact overall wellbeing. For example, consider rehabilitation technologies. A platform that delivers videos for teaching rehabilitation exercises (e.g., for chronic pain patients) may be successful at the interface level (being easy to use and providing helpful options), and even at the behavioral level (the person effectively performs the exercises regularly). But if the person does not “transfer” what is learned as part of rehabilitation to other aspects of their life (e.g., driving, sleeping), then it cannot be argued that the rehabilitation was successful, and improvement to overall wellbeing in life is unlikely to occur.

This sphere is also critical for identifying addiction. Most of us can recall someone who “had to delete an app because it was just taking up too much of their time.” They experienced over-engagement (ie. addiction). A casual game, for example, can be so need satisfying within the first few spheres, that at the life sphere, important activities get crowded out leading to drops in relatedness as human relationships are ignored, drops in autonomy as they feel less able to make decisions aligned with their values. Therefore, any technology wishing to claim it improves wellbeing, or even that it merely doesn't harm it, will need to measure at the life level.

While not all technology projects will aim for changes to long-term wellbeing, aiming to satisfy psychological needs has the potential to benefit all projects. In addition to increasing engagement and activity-specific outcomes, doing so may have positive collateral effects, for example by removing causes of stress, and increasing overall psychological need satisfaction in people's lives. Even if these improvements are not easily measurable or causally attributable to any one technology, they will still be contributing to a cumulative effect that could increase individual or even societal wellbeing measurably over time.

### SOCIETY—beyond the user experience

The sixth sphere in the model is largest in scope and is the only one to step beyond the *user* experience. Societal wellbeing may be affected by the use of a technology both directly and indirectly. Within this sphere, ethical issues regarding impact of an economic and environmental nature may become relevant. For example, a well-designed self-driving car may promote greater wellbeing and life satisfaction for many users. Yet the collateral impact of such cars on the livelihoods of the millions who survive off of driving taxis, buses and trucks can only be revealed at a societal level of analysis because this impact goes beyond the users of this technology. In fact, as technology penetrates social infrastructures, downstream effects are often multiple and interactive. This level of societal impact requires the consideration of interdependent factors, and therefore, will be, by far, the most difficult to accurately assess and will require multidisciplinary collaboration and new methods.

We now move to a discussion of evaluation measures for implementation of the model in practice.

## Evaluation measures

### Introduction to measures of psychological need satisfaction

In this section we review a number of validated instruments that can be used directly or adapted in order to measure the user experience of autonomy, competence and relatedness within the various spheres described by the METUX model.

The instruments described herein have been used in various contexts, for example to assess to what extent a medical professional (Williams et al., 2009), healthcare intervention (Williams et al., 2011; Teixeira et al., 2012) or a procedure (Ng et al., 2012) supports autonomy. SDT researchers have also used these instruments to evaluate the impact of coaches, teachers, education systems and learning technologies (Chen and Jang, 2010; Bartholomew et al., 2011). Decades of research provide evidence that psychological need-support within these environments has a significant impact on domain-specific (e.g., health, work and education) outcomes.

**Figure 4:**
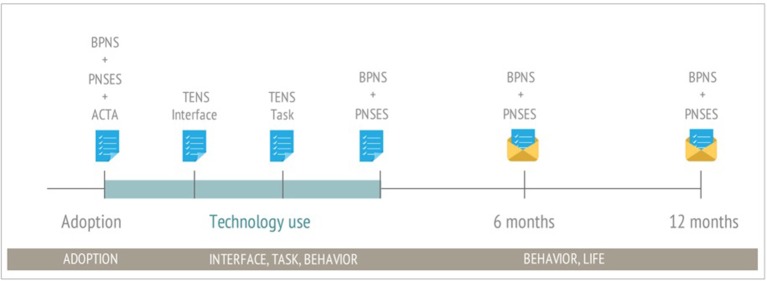
Evaluation timeline example – Example timeline of a wellbeing-supportive technology project highlighting the points at which SDT-based measures might be used for evaluation. METUX spheres are listed along the bottom. The PNSES is specific to exercise but would be replaced by an adaptation to the behavior domain relevant to the project. The PNSES and BPNS are given at baseline, 3, 6, and 12 months to show change over time, a common approach for psychology studies.

We show how these measures can be adapted to evaluate technological environments. The intention is to assist designers in measuring need-satisfaction related to their designs such that they can make iterative improvements that result in increased engagement, motivation and wellbeing, as has been done in other domains. This is precisely how SDT researchers have worked with game designers to increase user engagement in digital games (see Rigby and Ryan, 2011; Przybylski et al., 2014). Figure 4 shows an example of how SDT-based measures might be incorporated along the timeline of a wellbeing-supportive HCI project.

For three of the spheres we propose novel adaptations of existing SDT-based questionnaires adapted for the technology context. These are provided in Appendices 1–4. References to the literature available on the SDT-based measures discussed, along with links to many instruments, are available on the Self-determination theory website (www.selfdeterminationtheory.org).

### Measures for technology adoption

The Self-Regulation Questionnaire (SRQ) identifies types of motivation (autonomous vs. controlled) via questions concerning the regulation of a particular behavior (e.g., exercising regularly) or class of behaviors (e.g., engaging in religious behaviors) (Ryan and Connell, 1989). Therefore the SRQ can be readily adapted to assess motivation for the adoption of a technology, essentially focusing on the “why” of purchase/usage intentions. The questionnaire results range from amotivated, to controlled, to autonomous, with distinctions between intrinsic and extrinsic motivation as elaborated previously in Figure 2.

We have also argued that adoption will be mediated by anticipated competence and for this we recommend an adaptation of the Perceived Competence Scale (PCS). The original PCS includes 4 questions, and, in the context of technology adoption, only two would be relevant. These two can be added to an adapted SRQ to form a basic technology adoption questionnaire based on psychological need satisfaction. We have created such an adaptation, “Autonomy and Competence in Technology Adoption (ACTA)” and included it in Appendix 1. An initial validation of the ACTA is described in Appendix 5.

There has been considerable research into the adoption, intended use, and acceptance of technologies which has focused on other factors including demographic characteristics, traits, or variables to do with a specific domain. The specific research questions relating to a particular technology project will determine which measures are best suited to that project, however, we provide a measure based on psychological need satisfaction in order to provide a complete and theoretically consistent approach to the evaluation of technologies at all levels.

### Measures relating to the interface

Among the most common measures for evaluating a technology interface are questionnaires like the System Usability Scale (Bangor et al., 2008). While usability measures can be useful in identifying *obstacles* to engagement, high usability does not necessarily predict high engagement or positive experience (Febretti and Garzotto, 2009). In contrast, the PENS (Ryan et al., 2006) is a validated 21-item SDT-based questionnaire that has been shown to predict engagement and enjoyment. The PENS has been used to assess the experience of need satisfaction and user experience in video game contexts and has been refined in its ongoing use (see Rigby and Ryan, 2011). It assesses both the degree to which the user experiences mastery of the interface, need satisfaction during use, and qualities such as immersion and includes a number of questions only relevant to gaming that can be excluded for adaptation to other technologies.

We provide a complete adaptation of the PENS for non-game technologies, which we call the TENS-Interface (Technology-based Experience of Need Satisfaction–Interface) as Appendix 2. Validation data for the TENS-Interface is included in Appendix 5.

### Measures relating to the task

Because the PENS was developed for use in video games, for which, uniquely, the technology itself provides the activity it supports, the PENS also evaluates need satisfaction within the task sphere. As such, we have been able to adapt a task-based questionnaire from the PENS and provide this Technology-based Experience of Need Satisfaction-Task (TENS-Task) instrument as Appendix 3. The TENS-Task can be used to measure psychological need satisfaction provided by engagement with technology-supported tasks. Validation data for the TENS-Task is included in Appendix 5.

### Measures relating to the behavior domain

As discussed earlier, a technology generally mediates or supports a behavior in ways that are more or less satisfying to an individual's psychological needs. The SDT literature provides numerous examples of validated questionnaires for specific behavior domains including exercise, diet improvement, parent-child interaction and learning. For example, the Psychological Need Satisfaction in Exercise Scale (PNSES) (Wilson et al., 2006) measures perceived psychological need satisfaction when doing exercise and would therefore serve as a measure of need satisfaction at the behavior level for an exercise technology.

However, in many cases there will not already be a questionnaire adapted to the specific behavior in question. In this case, we recommend that the general Basic Psychological Needs Satisfaction questionnaire (BPNS; Chen et al., 2014) or General Self-Regulation Questionnaire be adapted to the context (much in the way existing domain-specific questionnaires were developed). For examples, see the development of the above-mentioned PNSES (Vlachopoulos and Michailidou, 2006) or an adaptation for the work domain (Broeck et al., 2010). Separate to measures of need satisfaction, projects are also likely to include domain-specific outcome measures.

### Measures relating to life

The TENS-Life (Technology Effects on Need Satisfaction in Life) scale (Appendix 4) is introduced as a measure to identify the extent to which users believe a technology has had an impact on need satisfaction in their lives. With items such as “I spend more time on the technology than I feel I should” and “using the technology has made me feel a greater sense of belonging to a community” the TENS-Life provides a direct link between a technology and wellbeing in life allowing for the identification of autonomy frustrations that may relate to addictive patterns. Validation data is provided in Appendix 5.

Another approach to measuring changes to overall wellbeing is via standard wellbeing measures run pre- and post-use of a technology. This is particularly useful for technology-based psychology interventions. The Basic Psychological Need Satisfaction (BPNS) scales provide a theoretically consistent measure. However, other validated measures of wellbeing are also available, such as Ryff's Psychological Wellbeing Scales (Ryff, 1989), the MHC-SF (Lamers et al., 2011), the Flourishing Scale (Diener et al., 2009), or frameworks in which wellbeing is conceptualized as lying at the opposite end of a spectrum of mental illness (Huppert and So, 2013).

## Conclusions: toward technology design for flourishing

In this paper we have argued that the impact of a technology on the psychological experience and wellbeing of an individual can be better understood, empirically evaluated, and designed for, by targeting basic psychological needs as defined by Self-determination Theory. We present a model for bridging SDT theory to technology design practice which we refer to as METUX (Motivation, Engagement & Thriving in User Experience). In order to ensure a sufficiently broad view of wellbeing (i.e., one that includes eudaimonia and accounts for addiction) the model posits that psychological needs be considered at five different separable spheres of analysis, including: at the point of technology *adoption*, during interaction with the *interface*, as a result of engagement with technology-specific *tasks*, as part of the technology-supported *behavior*, and as part of an individual's *life* overall. These five spheres of experience sit within a sixth, *society*, which encompasses both direct and collateral effects of technology use as well as *non*-user experiences.

We present examples of existing SDT-based measures, as well as introduce four new measures that can be used to evaluate need satisfaction at the five levels. According to research, in addition to predicting impact on wellbeing, motivation and sustained engagement with technology, SDT measures also predict the fulfillment of domain-specific outcomes (such as health or educational outcomes) making SDT an ideal basis for understanding and improving other common goals within technology projects.

Of course, a number of limitations should be noted. The measures presented are initial iterations that will require more thorough validation and refinement in response to usage over time. Moreover, the spheres themselves are approximations and other delineations may very well prove more useful overall or within different contexts. Finally, SDT, while a mature theory with robust support, remains a psychological theory open to ongoing interrogation. Further research on all fronts (with regard to the measures, the HCI implementation and the psychological basis in the technology context) is required and it is our hope that the theory and measures provided herein can form a useful starting point.

Our intention is that the model and instruments provided will enable technology designers to evaluate their technologies for wellbeing impact, and allow HCI researchers tools and theory upon which to improve. In this way, as a community we may iterate collectively toward a future in which all technologies are better designed to support psychological wellbeing and human potential.

## Ethics statement

This study was carried out in accordance with the recommendations of the Australian Code for the Responsible Conduct of Research and the National Statement on Ethical Conduct in Human Research. The protocol was approved by the Human Research Ethics Committee at Australian Catholic University (review number 2017-21516). All subjects gave informed consent in accordance with the Declaration of Helsinki.

## Author contribution

All authors listed have made a substantial, direct and intellectual contribution to the work, and approved it for publication.

### Conflict of interest statement

The authors declare that the research was conducted in the absence of any commercial or financial relationships that could be construed as a potential conflict of interest.
