# Supplementary material for: Designing for Motivation, Engagement and Wellbeing in Digital Experience
Source: Front Psychol. 2018 May 28;9:797. doi: 10.3389/fpsyg.2018.00797 (PMC5985470; doi:10.3389/fpsyg.2018.00797)
Supplement: Supplementary file 1 [file Presentation_1.PDF]

Appendix 1 – Autonomy and Competence in Technology Adoption Questionnaire (ACTA)

The ACTA questionnaire concerns why people adopt use of a technology (ie. download an app, register with a website, purchase a wearable device, etc.) and the extent to which they perceive they will be competent to use it. The ACTA is based on the Self-Regulation Questionnaire scales devised for other domains (ie. exercise, learning and healthcare) and the Perceived Competence Scales, both found on the self-determination theory web site.

The wording of the ACTA can be adapted to identify the specific technology in question. For example the text "decided to use" can be replaced with "downloaded", "purchased", or "registered for" as appropriate. Likewise, the term "technology" can be replaced with "app", "website", or the technology name, etc.

General Scoring Information for the ACTA. For the first set of questions, based on the SRQ scales, the responses on the autonomous items are averaged to form the autonomous regulation score for the target behavior and the responses on the controlled items (external and introjected) are averaged to form the controlled regulation score for the target behavior. For other SRQ versions, these two subscale scores are often used separately, but at times they have been combined into a Relative Autonomy Index (RAI) by subtracting the average for Controlled Regulation from the average for Autonomous Regulation. For the second set of questions (adapted from the PCS), a person's score is simply calculated by averaging his or her responses on the two items.

The Scale

There are a variety of reasons why people choose to purchase or start using a technology. Please consider the following and indicate how true each of these reasons is for you. The scale is:

1	2	3	4	5
not at all true		somewhat true		very true

Self-regulation

I decided to start using the technology because:

1. Other people want me to use it. *(external)*
2. I expected it will be interesting to use. *(intrinsic)*
3. I believe it could improve my life. *(identified)*
4. It will help me do something important to me. *(identified)*
5. I want others to know I use it. *(introjected)*
6. I will feel bad about myself if I didn't try it. *(introjected)*
7. I think it would be enjoyable. *(intrinsic)*
8. I am required to use it (eg. by my job, school, research study). *(external)*
9. It is going to be of value to me in my life. *(identified)*
10. It is going to be fun to use. *(intrinsic)*
11. I feel pressured to use it. *(external)*
12. It will look good to others if I use it. *(introjected)*

Perceived Competence

1. I feel confident that I'll be able to use the technology effectively.
2. The technology will be easy for me to use.

Appendix 2 – Technology-based Experience of Need Satisfaction – Interface questionnaire (TENS-Interface)

Administration Guidelines:

- Respondents typically rate their level of agreement to each item using a 5-point Likert scale (1= Do Not Agree, 5=Strongly Agree);
- All items are weighted equally in scoring;
- Items are randomized in their order when presented to participants;
- Reverse-scored items are indicated by "(-)";
- Questions are framed by the following stem:
  "Reflect on your experience using the technology and rate your agreement with the following statements:" in which "the technology" can be replaced by a more specific name (eg. "the app" or "FitnessPal") OR a specific interface component being assessed (eg. "the community forum", "the video conferencing tool") throughout the questionnaire.

Competence

1. I feel very capable and effective at using the technology.
2. I feel confident in my ability to use the technology.
3. Learning how to use the technology was difficult. (-)
4. I found the interface and controls confusing. (-)
5. It wasn't easy to use this technology. (-)

Autonomy

6. The technology provides me with useful options and choices
7. I can get the technology to do the things I want it to.
8. I feel pressured by the technology. (-)
9. The technology feels intrusive (-)
10. The technology feels controlling. (-)

Relatedness (optional)

11. The technology helps me to form or sustain relationships that are fulfilling.
12. The technology helps me to feel part of a larger community.
13. The technology makes me feel connected to other people.
14. I don't feel close to other users of the technology. (-)
15. The technology doesn't support meaningful connections to others. (-)

Appendix 3 – Technology-based Experience of Need Satisfaction – Task questionnaire (TENS-Task)

Administration Guidelines:

- Respondents rate their level of agreement to each item using a 5-point Likert scale (1= Do Not Agree, 5=Strongly Agree);
- All items are weighted equally in scoring;
- Items are randomized in their order when presented to participants;
- Reverse-scored items are indicated by "(-)";
- Questions are framed by the following stem:
  "Reflect on your experience using the technology to [task description] and rate your agreement with the following statements:" in which "the technology" can be replaced by a more specific name (eg. "the app" or "FitnessPal") and "the task" or "engaging in the task" can be replaced with a description of the task in question (eg. "track your steps", "setting health goals") throughout the questionnaire.

Competence

1. I feel confident in my ability to [do the task].
2. It's easy to [do the task].
3. I find [doing the task] too challenging. (-)
4. I find the task too difficult to do regularly (-)

Autonomy

5. I feel pressured to [do the task]. (-)
6. I only do the task because I have to. (-)
7. I [do the task] because other people want me to (-)
8. I will feel guilty if I don't [do the task] (-)

Relatedness (optional)

9. Doing the task helps me to form or sustain relationships that are fulfilling.
10. Doing the task helps me to feel part of a larger community.
11. I don't feel close to other users when I engage in the task. (-)
12. Doing the task doesn't support meaningful connections to others. (-)

Appendix 4 – Technology Effects on Need Satisfaction in Life (TENS-Life)

Scale Description

The TENS-Life investigates the extent to which a user perceives that the use of a particular technology has had an influence on the satisfaction of basic psychological needs in their life. It is based on the validated Basic Psychological Need Satisfaction and Frustration Scale (Chen, Vansteenkiste, et al., 2015).

Administration Guidelines:

- Respondents rate their level of agreement to each item using a 5-point Likert scale (1= Not true at all, 5=Completely true);
- All items are weighted equally in scoring;

- Items are randomized in their order when presented to participants;
- Reverse-scored items are indicated by "(-)";
- Questions are framed by the following stem:
  "Below, we are going to ask about how using the technology has or hasn't effected your actual experiences of certain feelings in your life. Choose from 1 to 5 to rate your agreement with the following statements:" in which "the technology" can be replaced by a more specific name (eg. "the app" or "FitnessPal") throughout the questionnaire.

Autonomy

1. Now that I use the technology, I feel pressured to use it more often than I'd like. (-)
2. I spend more time on the technology than I feel I should. (-)
3. The technology ends up making me do things I don't want to do. (-)
4. The technology intrudes in my life. (-)

Competence

5. Using the technology has made me feel insecure about my abilities. (-)
6. Using the technology has made me feel less capable in my life. (-)
7. Using the technology has lowered my confidence. (-)

Relatedness

8. Using the technology has helped me feel a greater sense of belonging to a larger community
9. Using the technology has helped me feel close and connected with other people who are important to me.
10. Because of this technology, I feel closer to some others.

Appendix 5 – Validation of introduced measures

The ACTA, TENS-Interface, TENS-Task and TENS-Life introduced within the paper and as Appendices 1-4 are novel measures based on SDT-based questionnaires from other domains. To provide initial validation for these, we carried out a pilot validation study in which 400 participants in total (100 for each of four technologies) were asked to fill out each METUX questionnaire in reference to one of four possible technologies: Facebook, Google Docs, a music streaming service and a fitness band. Results showed satisfactory to good internal consistency for all questionnaires with alphas for subscales ranging from 0.66 to 0.88.

Specifically, we calculated Cronbach's alphas to test the internal consistency of the four questionnaires. Satisfactory internal consistency was seen in the Autonomy and Competence in Technology Adoption Questionnaire (ACTA) subscales with an alpha of 0.73 for the external sub-scale, 0.77 for the introjected sub-scale, 0.80 for the introjected subscale, and 0.82 for the intrinsic subscale.

For the three Technology-based Experience of Need Satisfaction questionnaires (Interface, Task and Life), satisfactory internal consistency was seen across subscales with alphas from 0.67 (autonomy) to 0.75 (relatedness) and 0.79 (competence) for TENS-Interface, and from 0.66 (relatedness), to 0.76 (autonomy) and 0.79 (competence) for TENS-Task. For the TENS-

Life questionnaire, good alphas were from 0.80 (autonomy) to 0.88 (relatedness and competence).

To further assess the validity of our model, we calculated the average correlation across the four technologies between the autonomy, competence, and relatedness subscales at each of the three spheres (Interface, Task, Life). Competence at the Interface correlated with competence at the Task an average of $r = 0.74$, and with competence in Life at $r = 0.56$, while competence at the Task and in Life correlated at $r = 0.57$. Autonomy at the Interface correlated an average of $r = 0.52$ with the Task, and $r = 0.61$ with Life, while autonomy in the Task and autonomy in Life correlated an average of $r = 0.69$ across the four technologies. Relatedness at the Interface correlated with the Task and Life at $r = 0.74$ on average, while relatedness in the Task and in Life correlated at $r = 0.64$. Correlations of this magnitude indicate that, as expected, the constructs are highly related and yet distinct at each sphere of the model, suggesting that these questionnaires can provide theoretically coherent, and novel information at each tier.

Additionally, we ran path analyses on the three TENS questionnaires to examine the pathways by which need satisfaction at the Interface, Task and in Life influence one another, and overall technology satisfaction. While a detailed discussion of these analyses is beyond the scope of this paper, the initial results indicated that need satisfaction at the Interface predicts need satisfaction in the Task and, often, need satisfaction in Life and overall satisfaction with the technology. Furthermore, since the model predicts engagement, successful technologies such as those tested would indeed be expected to show need satisfaction at Interface and Task, and these can be considered “gateways" to other satisfactions.

As we would expect, the relationships among these spheres and their links to overall satisfaction differed across technologies. For example, relatedness satisfaction in Life as a result of the use of Facebook predicted overall satisfaction with Facebook (β= 0.47), but the same was not true for Google Docs. Thus, we would expect the profile of direct and indirect effects to be different for each technology.

While this pilot validation provides promising early evidence in support of the new measures proposed, please note, a more complete validation study is planned that will explore and describe the relationships, provide more reliable evidence, and refine the measures.
